# Human population movement and behavioural patterns in malaria hotspots on the Thai–Myanmar border: implications for malaria elimination

**DOI:** 10.1186/s12936-019-2704-3

**Published:** 2019-03-08

**Authors:** Sayambhu Saita, Wirichada Pan-ngum, Suparat Phuanukoonnon, Patchara Sriwichai, Tassanee Silawan, Lisa J. White, Daniel M. Parker

**Affiliations:** 1 Department of Tropical Hygiene, Faculty of Tropical Medicine, Mahidol University, Bangkok, Thailand; 2 Mahidol-Oxford Tropical Medicine Research Unit, Faculty of Tropical Medicine, Mahidol University, Bangkok, Thailand; 3 Department of Social and Environmental Medicine, Faculty of Tropical Medicine, Mahidol University, Bangkok, Thailand; 4 Department of Medical Entomology, Faculty of Tropical Medicine, Mahidol University, Bangkok, Thailand; 5 Department of Community Health, Faculty of Public Health, Mahidol University, Bangkok, Thailand; 6 Centre for Tropical Medicine, Nuffield Department of Medicine, University of Oxford, Oxford, UK; 7 Department of Population Health and Disease Prevention, University of California, Irvine, USA

**Keywords:** Human population movement, Malaria hotspots, Thai–Myanmar border, Spatial epidemiology, Spatial demography

## Abstract

**Background:**

Malaria is heterogeneously distributed across landscapes. Human population movement (HPM) could link sub-regions with varying levels of transmission, leading to the persistence of disease even in very low transmission settings. Malaria along the Thai–Myanmar border has been decreasing, but remains heterogeneous. This study aimed to measure HPM, associated predictors of travel, and HPM correlates of self-reported malaria among people living within malaria hotspots.

**Methods:**

526 individuals from 279 households in two malaria hotspot areas were included in a prospective observational study. A baseline cross-sectional study was conducted at the beginning, recording both individual- and household-level characteristics. Individual movement and travel patterns were repeatedly observed over one dry season month (March) and one wet season month (May). Descriptive statistics, random effects logistic regressions, and logistic regressions were used to describe and determine associations between HPM patterns, individual-, household-factors, and self-reported malaria.

**Results:**

Trips were more common in the dry season. Malaria risk was related to the number of days doing outdoor activities in the dry season, especially trips to Myanmar, to forest areas, and overnight trips. Trips to visit forest areas were more common among participants aged 20–39, males, individuals with low income, low education, and especially among individuals with forest-related occupations. Overnight trips were more common among males, and individual with forest-related occupations. Forty-five participants reported having confirmed malaria infection within the last year. The main place of malaria blood examination and treatment was malaria post and malaria clinic, with participants usually waiting for 2–3 days from onset fever to seeking diagnosis. Individuals using bed nets, living in houses with elevated floors, and houses that received indoor residual spraying in the last year were less likely to report malaria infection.

**Conclusion:**

An understanding of HPM and concurrent malaria dynamics is important for consideration of targeted public health interventions. Furthermore, diagnosis and treatment centres must be capable of quickly diagnosing and treating infections regardless of HPM. Coverage of diagnosis and treatment centres should be broad, maintained in areas bordering malaria hotspots, and available to all febrile individuals.

**Electronic supplementary material:**

The online version of this article (10.1186/s12936-019-2704-3) contains supplementary material, which is available to authorized users.

## Background

Human population movement (HPM) and travel patterns are important with regard to infectious disease epidemiology. Infectious diseases such as malaria are heterogeneously distributed across landscapes, perhaps especially in low transmission settings. HPM can link sub-regions with varying levels of transmission, leading to the persistence of disease even in very low transmission settings [1, 2]. For example, very low transmission settings might achieve local elimination in the absence of being linked to high transmission settings via HPM [3, 4]. HPM has been suggested to be one factor in the failure to eliminate malaria during previous malaria eradication programs [3, 5, 6]. HPM data, when coupled with malaria epidemiological data, can help to identify potential “sources” and “sinks” of malaria parasites with direct implications for malaria control and elimination efforts [7, 8].

As in other low-transmission settings, malaria in the Greater Mekong Sub-region (GMS) is heterogeneous and patchy, with many sub-regions having little or no malaria transmission. The disease tends to cluster in border regions that have suitable environmental, ecological, socio-economic, and demographic characteristics that contribute to the persistence of malaria. For example, while malaria has been greatly reduced in Thailand over the last several decades, the disease continues to persist along international borders; one of the heaviest burdens has been along the Thai–Myanmar border. HPM within, to, and from this border area has been considered an important contributor to overall malaria epidemiology [9–11]. Moreover, travelling cross-border to Myanmar and/or into forest areas has been considered important with regard to the risk of malaria infection and the persistence of malaria along this international border [12–15].

Political unrest between ethnic and political groups in Myanmar has frequently led to significant cross-border population movements, and displaced minority populations have historically had increased risk of malaria infection. Human population expansions, connectivity, and environmental changes have all contributed to the risk of acquiring infection in a heterogeneous malaria landscape as well as the risk of reintroduction in places that have achieved elimination [11, 16–18].

Previous research on the space–time distributions of malaria along the Thai–Myanmar border indicated two persistent malaria hotspots of both *Plasmodium falciparum* and *Plasmodium vivax* incidence: one was in Tha Song Yang District, Tak Province, and another in the adjacent areas of Umphang District of Tak Province and Sangkhlaburi District of Kanchanaburi Province [19]. Malaria incidence has been decreasing, but remains heterogeneous; some sub-districts were near elimination levels, while most of sub-districts were in the pre-elimination level (< 1 case per 1000 people per year) [20]. As Thailand (and other nations of the GMS) has committed to eliminating malaria by the year 2030 [21], it will be important to have an understanding of the spatial demography (including human movement patterns) and epidemiology of endemic and surrounding areas that are prone to re-importation of parasites (e.g. with suitable environments and mosquito vector populations) [3, 22]. Thus, this study aimed to measure HPM patterns, associated predictors of travel, and HPM correlates of self-reported malaria (SRM) infections among people living within malaria hotspots on the Thai–Myanmar border.

## Methods

### Study design

This study began with a baseline cross-sectional study, recording both individual- and household-level characteristics. Repeated cross-sectional surveys that recorded individual movement and travel patterns were collected weekly over one dry season month (March) and one wet season month (May), with seasons defined based on Thai Meteorological Department classifications (described in more detail below).

### Study site

Previous work in the region showed two persistent malaria clusters at the sub-district level: Tha Song Yang Sub-district of Tak Province (Cluster I) and Nong Lu Sub-district of Kanchanaburi Province (Cluster II). Both sub-districts were purposively selected to representative malaria hotspots in this research. Here we use the term “hotspot” to indicate an area (i.e. district) with higher than normal malaria burden. The area is mountainous and partly covered by rainforests. Generally the climate of Thailand divided into three seasons: rainy or southwest monsoon season (mid-May to mid-October), winter or northeast monsoon season (mid-October to mid-February), and summer or pre-monsoon season (mid-February to mid-May) [23]. However, in the region along the border with Myanmar the monsoon rains are more intense than in other parts of Thailand. In 2017, cumulative rainfall in this region was 1000 to 1800 mm, average temperature was 26 to 27 °C.

**Fig. 1:**
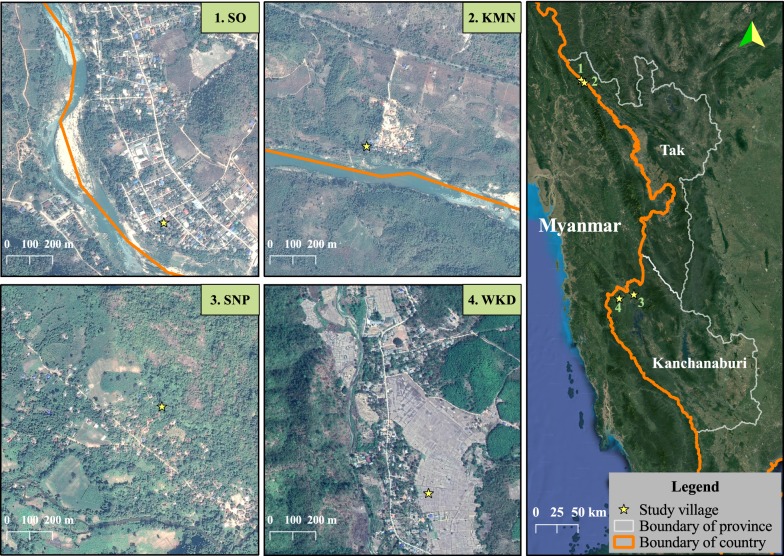
Study villages

**Fig. 2:**
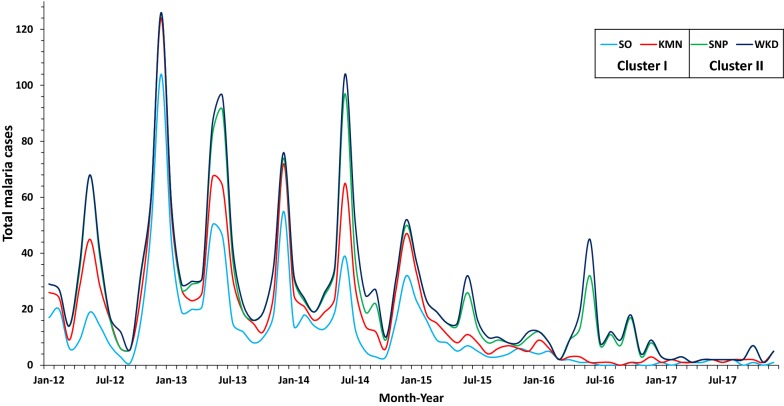
Counts of malaria cases. Counts include all species and both villagers and non-residents

The total population of the study sub-districts at the end of 2017 was 39,237 people. Two villages from each sub-district were chosen, with criteria being that they must have transmission reported ≥ 6 months per year. The selected villages in Cluster I were SO and KMN and in Cluster II were SNP and WKD. The villages are located on forest fringes, in hilly-to-lower hillslope areas. All study villages are located in the Thai side (≤ 10 km.) of the international border, adjacent to Kayin State of Myanmar (Fig. 1). Clinical malaria cases in the study sites exhibit similar seasonal patterns and decreased over time. Total malaria case numbers in Cluster II were higher than Cluster I (Fig. 2).

### Sample size

The sampling strategy was set up to capture normal movement patterns among people who live in malaria endemic villages on the Thai–Myanmar border. Persistent sub-district level clusters of malaria incidence were previously identified [19] and these clusters were targeted for this study. Four villages were chosen based on their location (within the identified persistent malaria clusters) and based on year-round transmission of malaria, according to local Vector Borne Disease Control Unit data (Fig. 2). The total population of the four selected villages in 2017 was 5073. Survey participants were selected by household. Previous work in the region has shown an average household size of approximately 5 individuals per house [24], leading to an estimated 1015 households across the four villages. Assuming that 50% of households will have a participant who has a trip during the study period, it would be necessary to sample 280 households in order to achieve 95% confidence level with a precision of 0.05.

### Data collection and analysis

The individual- and household-level data were collected using a questionnaire at the beginning of the study period, targeting the head of household and one other family member who engages in activities which potentially involve travelling into forest areas or outside of the village. The questionnaire comprised of (1) household characteristics; number of household members, roof materials, wall materials, floor structure, and receiving indoor residual spraying (IRS) in the last year, (2) general respondent characteristics; age, gender, educational level, Thai literacy (complete listening-speaking-reading-writing skills), forest-related occupations (rice paddy field, corn and sugar cane fields, rubber plantation, fruit orchard, agricultural laborers, forestry officer, livestock), and monthly income, and (3) history of malaria infection, personal protection, and treatment behaviours. Malaria infection in this region is diagnosed in malaria posts (by rapid diagnostic test), malaria clinics, or hospitals (both by microscopy).

Movement and travel patterns were observed using a standardized daily movement form for a complete month in March (dry season) and again in May (wet season) 2018. Data included in the form included whether or not the head of household or other household participant made a trip within the previous week, whether the trip was within Thailand or to Myanmar, visited areas (i.e. other villages or forest), and the type of trip (daytrip or overnight). The form was repeatedly filled in at the end of each week (on Sunday) for four consecutive weeks during both of the study months.

Descriptive statistics (frequency, percentage, mean, and standard deviation) were used to describe travel and movement patterns. Random effects logistic regressions were used to assess potential predictors (previously listed covariates) of travel, with different regressions by travel type (day or overnight trip), times (wet or dry season), and location (within Thailand or to Myanmar, to another village or to a forest area). Random intercepts were used to account for repeat observations within individual participants and within households. Trips were aggregated at the week level and an offset was used to account for the number of trips within a week. Subsequent logistic regressions were used to determine factors that were associated with malaria infections. Model adjusted odds ratios and 95% confidence interval (AOR [95% CI]) were calculated to assess the magnitude and statistical significance of potential predictors of travel and self-reported malaria infections.

## Results

### Summary stats of human population movement (HPM) patterns

**Fig. 3:**
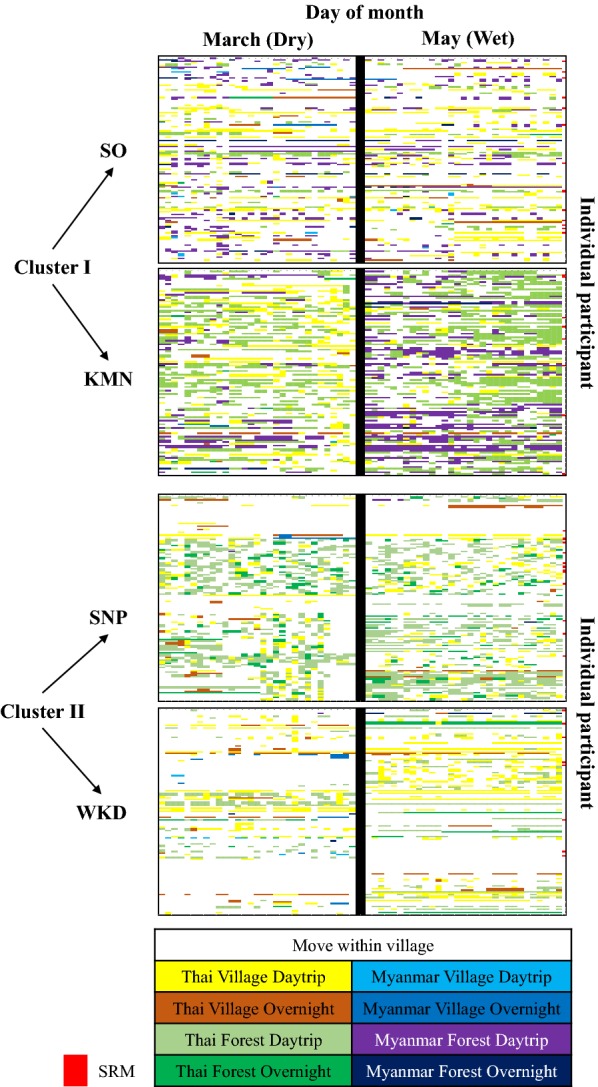
Individual travel patterns by study village, travel destination, and travel type

In total, 526 individuals from 279 households in the two cluster areas were included in the full prospective observational study, of which 249 individuals from 140 households were in the Cluster I and 277 individuals from 139 households were in the Cluster II. The individual movement data indicate daily travel destinations and travel types among individual participants by study village (Fig. 3). In both clusters, trips were more common in the dry season.

In Cluster I during the dry season, 10.04% of all participants did not leave their village while in the wet season 16.87% of all participants never left their village (Table 1). Seventy-six percent of all participants made at least one trip within the Thai side, 69.88% made trips to other villages, 72.29% made trips to forest areas, 88.35% made daytrips, and 19.28% made overnight trips in the dry season. Trips to Myanmar were similar in both dry and wet seasons (≅ 44.00%). In the wet season, more days were spent (on average, indicated by ($\overline{d}$) in Table 1) on each trip type, with the exception being trips to other villages.

**Table 1:** Proportion of study participants who made a trip, by trip destination (on the Thai versus Myanmar side of the border or to another village versus forested area) and time (daytrips versus overnight trips)

Characteristic of movement	Cluster I (n = 249)				Cluster II (n = 277)			
	Dry		Wet		Dry		Wet	
	%	$\overline{d}$	%	$\overline{d}$	%	$\overline{d}$	%	$\overline{d}$
Move within village	10.04		16.87		31.05		37.18	
Move-out to others	89.96		83.13		68.95		62.82	
Visiting side								
Thai	76.31	7.18	73.09	7.70	65.34	6.86	62.82	8.84
Myanmar	43.78	2.28	44.18	4.27	6.50	0.23	1.08	0.08
Visiting place								
Village	69.88	3.68	49.80	2.94	55.23	2.28	50.18	3.37
Forest	72.29	6.38	66.67	9.04	48.74	4.28	49.10	5.55
Type of trip								
Daytrip	88.35	9.12	81.53	11.23	64.62	5.58	60.29	7.18
Overnight	19.28	0.95	10.04	0.74	39.35	1.52	25.99	1.74

The proportions do not add up to 100% because individual participants engaged in multiple trip types (e.g. individuals are capable of both making trips to the Thai side and the Myanmar side). ($\overline{d}$) indicates the mean number of days by trip type

In Cluster II, 65.34% of all participants made trips within the Thai side, 6.50% made trips to the Myanmar side, 55.23% made trips to other villages, 64.62% made daytrips, and 39.35% made overnight trips during the dry season. Trips to forest areas were similar in both seasons (≅ 49.00%). During the wet season the number of days spent on each trip type were greater than during the dry season, with the exception of trips to Myanmar **(**Table 1). However, most participant spent 2–4 days on each trip and trips were more frequent in the dry season (see Additional file 1).

### Logistic regressions for odds of having a trip, by trip destination and duration

#### Logistic regressions by travel destinations

##### Predictors for the odds of travelling to Myanmar

**Fig. 4:**
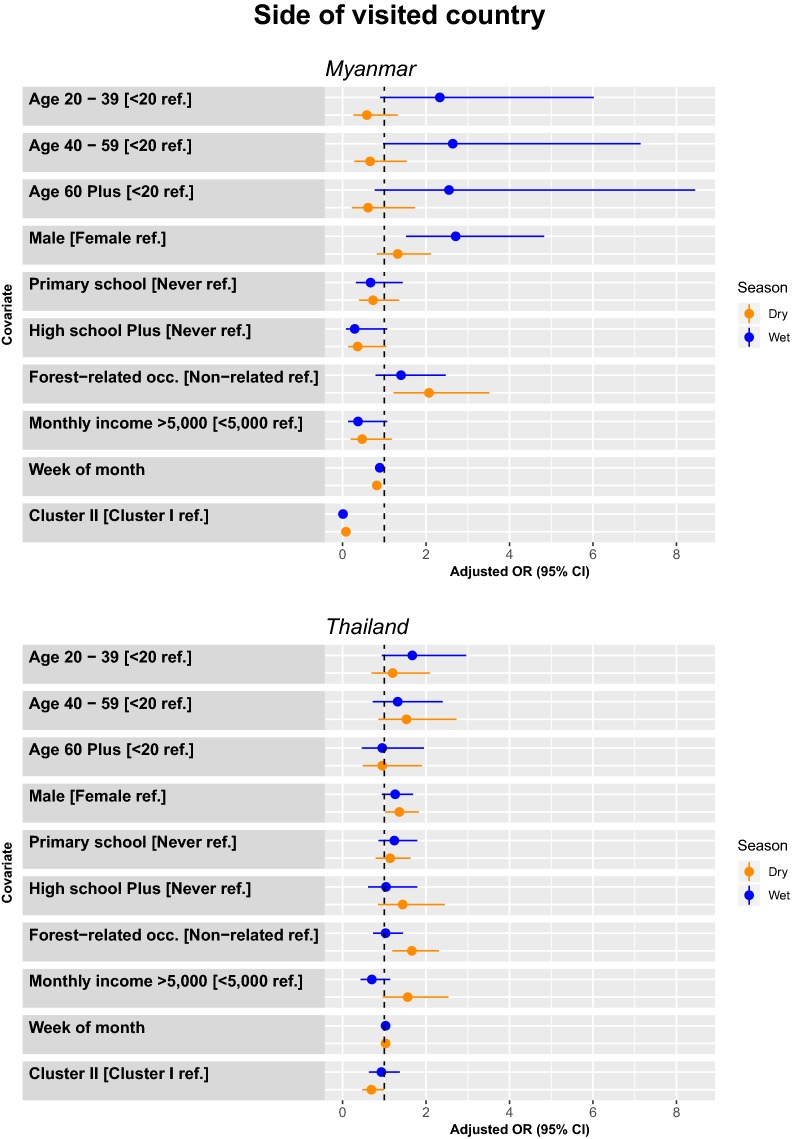
Model adjusted odds ratios and confidence intervals for travel to either Myanmar (top panel) or within Thailand (bottom panel) by season (wet season is blue, dry season is orange)

During the dry season, there were more trips among people who have forest-related occupations (2.07 [1.22–3.52]), in the earlier weeks of the month (0.82 [0.73–0.93]), and in Cluster I. During the wet season, there were more trips among males (2.71 [1.52–4.83]) and among participants from Cluster I (Fig. 4).

##### Predictors for the odds of travelling within Thailand

Males (1.37 [1.02–1.82]) and study participants with forest-related occupations (1.66 [1.19–2.31]) were more likely to have trips within Thailand in particular in the dry season (Fig. 4).

##### Predictors for the odds of travelling to a forest area

**Fig. 5:**
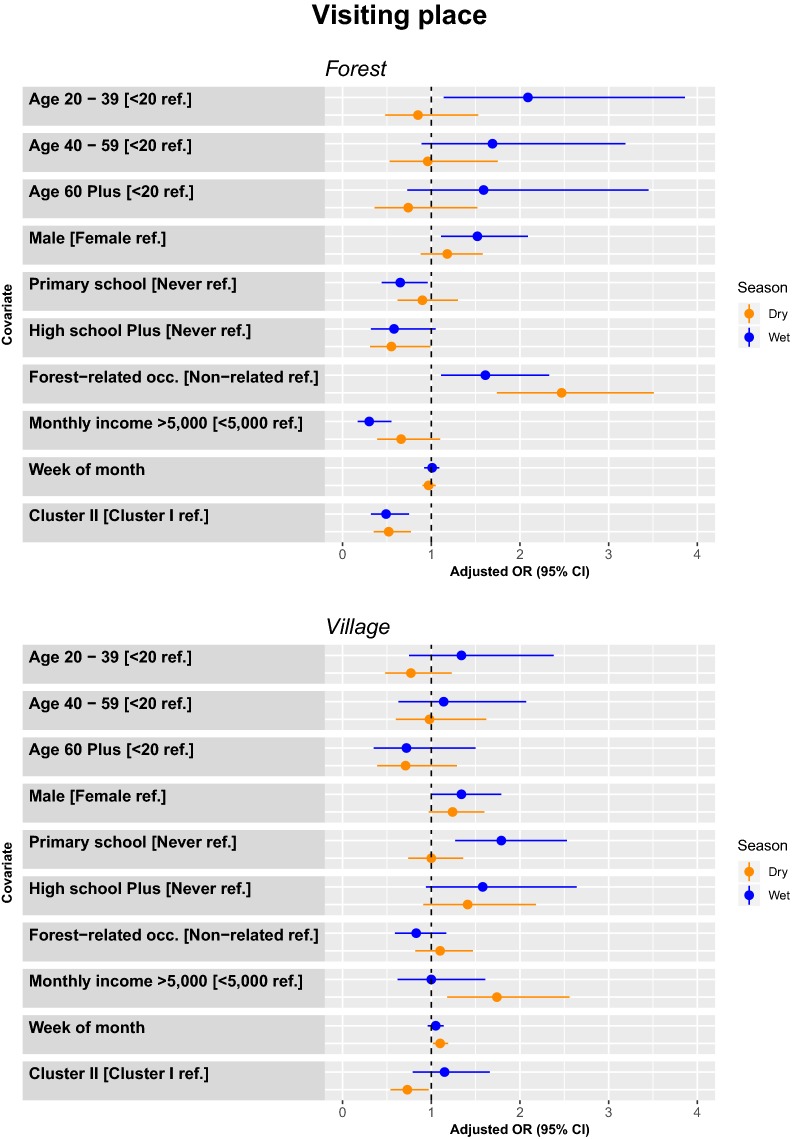
Model adjusted odds ratios and confidence intervals for travel to either forest area (top panel) or other villages (bottom panel) by season (wet season is blue, dry season is orange)

During the dry season, there were more trips among people who have forest-related occupations (2.47 [1.74–3.51]), and among participants from Cluster I. In comparison to those with no education, those with ≥ high school education had half the odds of visiting the forest (0.55 [0.31–0.98]). During the wet season, there were more trips among people in the 20–39 year age group (2.09 [1.14–3.86]), male (1.52 [1.11–2.09]), forest-related occupations (1.61 [1.11–2.33]), and among participants from Cluster I. Trips to the forest were more common among people who had no education, and who had lower monthly income. In comparison to those with no education, individuals with primary school education had a 45% decrease in odds of making a forest visit (0.65 [0.44–0.96]), and those with monthly income > 5000 THB had 69% decrease in the odds of making a trip to the forest (0.31 [0.17–0.55]) (Fig. 5).

##### Predictors for the odds of travelling to another village

During the dry season, there were more trips among people who had monthly income > 5000 THB (1.74 [1.18–2.56]) and among participant in Cluster I. During the wet season, there were more trips among males (1.34 [1.01–1.80]) and individuals with primary school education (1.79 [1.27–2.53]) (Fig. 5).

#### Logistic regression by type of trip (overnight or daytrip)

##### Overnight trips

**Fig. 6:**
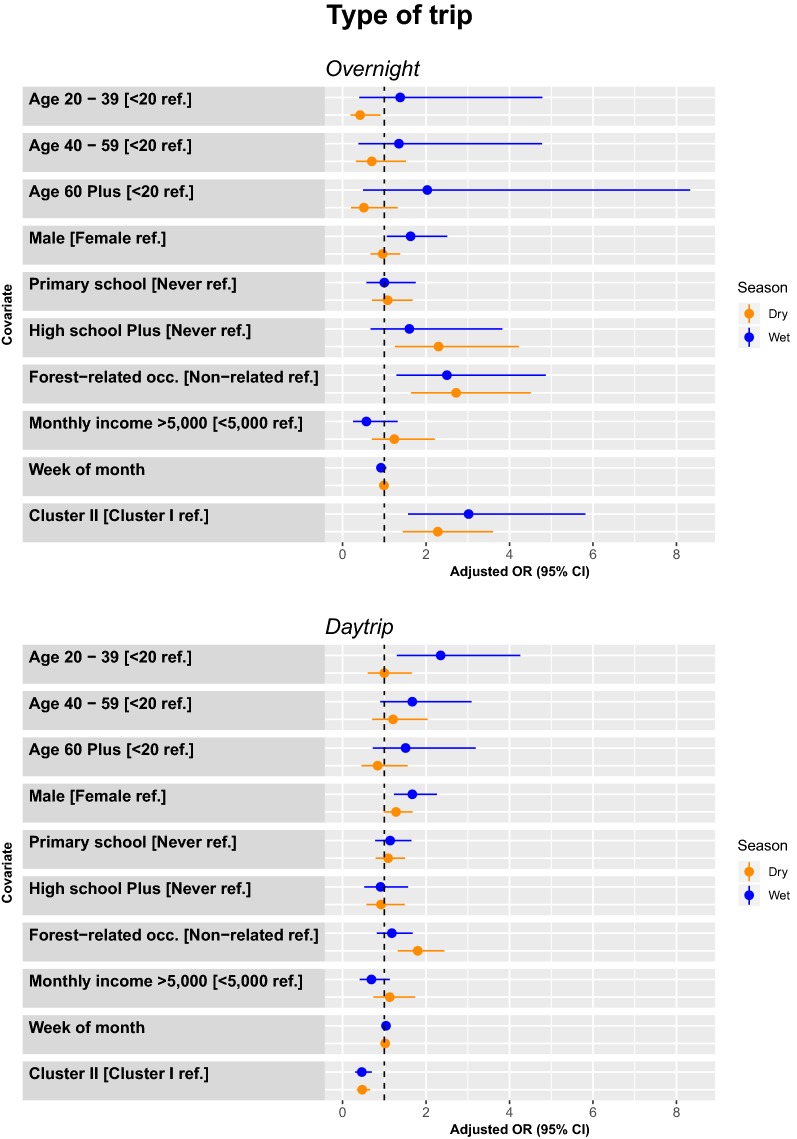
Model adjusted odds ratios and confidence intervals for travel by either overnight (top panel) or daytrip (bottom panel) by season (wet season is blue, dry season is orange)

During the dry season, there were more overnight trips among people age < 20 years compared to those age 20–39 (0.42 [0.19–0.91]), those with ≥ high school (2.30 [1.25–4.23]), forest-related occupations (2.72 [1.64–4.51]), and among participant in Cluster II. During the wet season, there were more trips among males (1.63 [1.06–2.51]) forest-related occupations (2.50 [1.29–4.87]), and among participants in Cluster II (Fig. 6).

##### Daytrips

During the dry season, there were more trips among people with forest-related occupations (1.80 [1.32–2.44]), and among participants in Cluster I. During the wet season, there were more trips among people with primary school education (2.35 [1.30–4.26]), males (1.67 [1.23–2.26]), and among participants in Cluster I (Fig. 6).

### Associations between duration of trip and SRM, by trip destination and type

The adjusted odds ratio and 95% confidence interval (AOR [95% CI]) indicated the increasing of the day in each trip in the dry season were more likely to increase the risk of malaria infection, especially trips to Myanmar (1.11 [1.03–1.17]), forested areas (1.08 [1.03–1.13]), and overnight trips (1.10 [1.02–1.19]). However, trips within Thailand and daytrips in the dry season also showed an association with SRM (1.05 [1.01–1.09] and 1.06 [1.02–1.11], respectively) (Table 2).

**Table 2:** Association between HPM and SRM

Characteristics	n	SRM (%)	OR [95% CI]	AOR [95% CI]
Side of country				
Within Thai side				
Duration				
Dry season		7.01 ± 7.57	1.04 [1.01–1.08]	1.05 [1.01–1.09]
Wet season		8.30 ± 5.57	1.00 [0.97–1.04]	1.00 [0.95–1.03]
Cluster area				
Cluster I	249	22 (8.84)	Ref.	Ref.
Cluster II	277	23 (8.30)	0.93 [0.51–1.72]	0.96 [0.52–1.77]
Myanmar side				
Duration				
Dry season		1.49 ± 4.06	1.07 [1.01–1.13]	1.11 [1.03–1.17]
Wet season		2.07 ± 5.49	0.99 [0.94–1.05]	0.95 [0.88–1.02]
Cluster area				
Cluster I	249	22 (8.84)	Ref.	Ref.
Cluster II	277	23 (8.30)	0.93 [0.51–1.72]	1.08 [0.54–2.14]
Visiting place				
Forests				
Duration				
Dry season		5.28 ± 6.87	1.06 [1.02–1.10]	1.08 [1.03–1.13]
Wet season		7.20 ± 8.75	1.00 [0.96–1.03]	0.96 [0.92–1.01]
Cluster area				
Cluster I	249	22 (8.84)	Ref.	Ref.
Cluster II	277	23 (8.30)	0.93 [0.51–1.72]	0.95 [0.50–1.80]
Other villages				
Duration				
Dry season		3.23 ± 5.25	1.03 [0.98–1.08]	1.03 [0.98–1.09]
Wet season		3.17 ± 5.83	1.01 [0.96–1.06]	0.99 [0.94–1.05]
Cluster area				
Cluster I	249	22 (8.84)	Ref.	Ref.
Cluster II	277	23 (8.30)	0.93 [0.51–1.72]	0.96 [0.52–1.78]
Type of trip				
Overnight				
Duration				
Dry season		1.25 ± 3.10	1.08 [1.01–1.16]	1.10 [1.02–1.19]
Wet season		1.27 ± 4.20	0.99 [0.91–1.07]	0.96 [0.88–1.05]
Cluster area				
Cluster I	249	22 (8.84)	Ref.	Ref.
Cluster II	277	23 (8.30)	0.93 [0.51–1.72]	0.90 [0.48–1.67]
Daytrip				
Duration				
Dry season		7.25 ± 7.55	1.05 [1.01–1.09]	1.06 [1.02–1.11]
Wet season		9.10 ± 8.81	1.00 [0.97–1.04]	0.98 [0.94–1.02]
Cluster area				
Cluster I	249	22 (8.84)	Ref.	Ref.
Cluster II	277	23 (8.30)	0.93 [0.51–1.72]	1.06 [0.55–2.02]

### Associations between individual- and household-characteristics and SRM

**Fig. 7:**
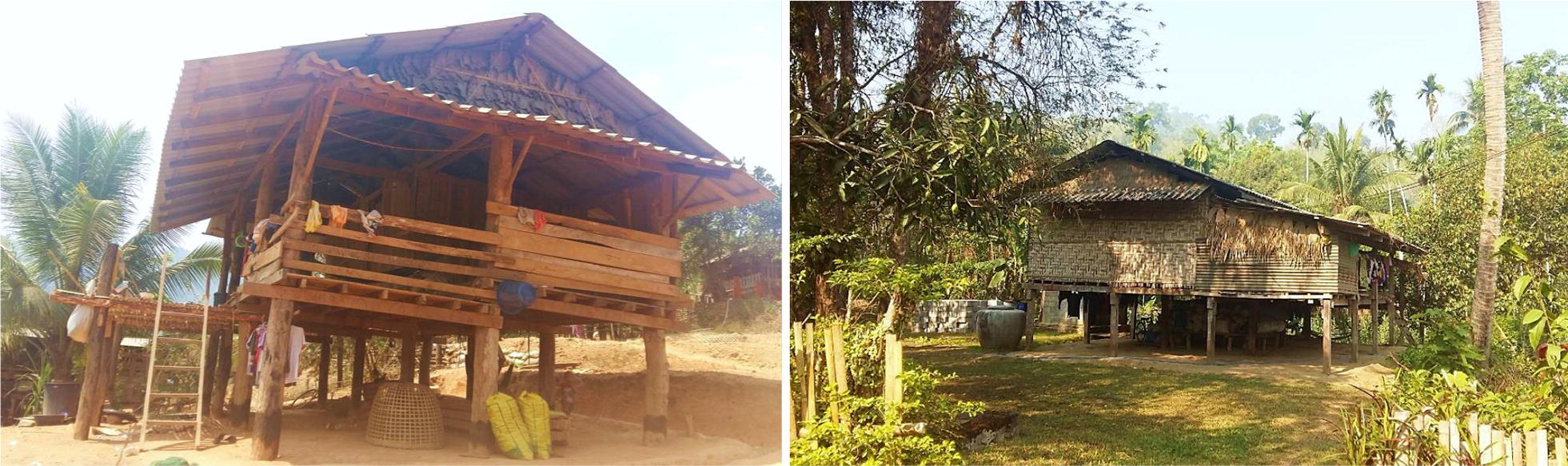
Example of elevated housing in study villages

There were 45 participants who reported having confirmed malaria infection(s) within the last year (see Additional file 2 for individual characteristics by study village). The majority of SRM were among participants aged 20 to 39 (42.22%), females (51.11%), those with no education (55.56%), who were not literate in Thai (73.33%), with forest-related occupations (64.44%), and with monthly income < 5000 THB (91.11%). Individuals living in houses with elevated floors (e.g. on stilts, see in Fig. 7) (0.27 [0.08–0.88]) and houses which received IRS in the last year (0.32 [0.11–0.92]) were less likely to report malaria infections (Table 3).

**Table 3:** Associations between individual and household characteristics and SRM

Characteristics	n	SRM (%)	OR [95% CI]	AOR [95% CI]
Individual level				
Age				
$\overline{x}$ ± SD	40.10 ± 14.79		0.97 [0.95–0.99]	0.95 [0.88–1.01]
< 20	44	10 (22.73)	Ref.	Ref.
20–39	214	19 (8.88)	0.33 [0.14–0.77]	0.49 [0.11–2.06]
40–59	212	12 (5.66)	0.20 [0.08–0.51]	0.71 [0.06–8.31]
≥ 60	56	4 (7.14)	0.26 [0.08–0.90]	2.11 [0.06–76.44]
Gender				
Female	318	23 (7.23)	Ref.	Ref.
Male	208	22 (10.58)	1.52 [0.82–2.80]	1.35 [0.66–2.76]
Educational level				
Never	329	25 (7.60)	Ref.	Ref.
Primary school	140	16 (11.43)	1.57 [0.81–3.04]	1.41 [0.49–4.07]
High school or above	57	4 (7.02)	0.31 [0.31–2.74]	1.02 [0.21–4.91]
Thai literacy (4 skills)				
No	381	33 (8.66)	Ref.	Ref.
Yes	145	12 (8.28)	1.05 [0.53–2.10]	0.47 [0.16–1.44]
Occupation				
Non-related forest	196	16 (8.16)	Ref.	Ref.
Related forest	330	29 (8.79)	1.08 [0.57–2.05]	1.60 [0.69–3.71]
Monthly income				
< 5000	460	41 (8.91)	Ref.	Ref.
> 5000	66	4 (6.06)	0.66 [0.23–1.90]	0.75 [0.22–2.62]
Household level				
Number of household’s member				
$\overline{x}$ ± SD	4.95 ± 2.62		1.09 [1.01–1.18]	1.08 [0.97–1.19]
Roof				
Tiles	217	16 (7.37)	Ref.	Ref.
Leaves/Canvas	102	11 (10.78)	1.52 [0.68–3.40]	1.55 [0.58–4.17]
Metal sheets	207	18 (8.70)	1.20 [0.59–2.41]	1.28 [0.55–2.95]
Wall				
Cements	100	9 (9.00)	Ref.	Ref.
Bamboos	213	18 (8.92)	0.99 [0.43–2.27]	1.72 [0.45–6.52]
Woods	213	17 (7.98)	0.88 [0.38–2.04]	1.65 [0.47–5.82]
Floor				
Ground floor	130	15 (11.54)	Ref.	Ref.
Elevated floor	396	30 (7.58)	0.63 [0.33–1.21]	0.27 [0.08–0.88]
Received IRS in the last year				
No	38	6 (15.79)	Ref.	Ref.
Yes	448	39 (7.99)	0.46 [0.18–1.18]	0.32 [0.11–0.92]
Cluster area				
Cluster I	249	22 (8.84)	Ref.	Ref.
Cluster II	277	23 (8.30)	1.07 [0.58–1.97]	0.66 [0.25–1.74]

AOR were adjusted from both individual and household levels

### Association between personal protection measures and SRM

Personal protection measures among individuals with SRM were mainly using long-sleeved shirts and pants (12.17%) and smoke or fire (11.02%) as protection against mosquito bites. Approximately 16.98% did not used bed nets and 21.05% did not sleep under bed net in the last night were found in SRM. Individuals who reported using bed nets had a 64% decrease in the odds of also having a malaria infection in the past year (0.36 [0.18–0.72]) (Table 4).

**Table 4:** Associations between SRM and personal protective measures

Protective measures	n	SRM (%)	OR [95% CI]	AOR [95% CI]
Untreated or treated nets				
No	106	18 (16.98)	Ref.	Ref.
Yes	420	27 (6.43)	0.34 [0.18–0.64]	0.36 [0.18–0.72]
Mosquito coil or repellent				
No	363	33 (9.09)	Ref.	Ref.
Yes	163	12 (7.36)	0.80 [0.40–1.58]	0.91 [0.42– 2.01]
Wearied long shirt and long pants				
No	337	22 (6.53)	Ref.	Ref.
Yes	189	23 (12.17)	1.98 [1.07–3.67]	1.77 [0.89–3.51]
Smoke or fire				
No	399	31 (7.77)	Ref.	Ref.
Yes	127	14 (11.02)	1.47 [0.76–2.86]	1.71 [0.84–3.49]
Never used or others				
No	403	39 (9.68)	Ref.	Ref.
Yes	123	6 (4.88)	0.48 [0.20–1.16]	0.50 [0.19–1.35]
Sleep under bed net in last night				
No	57	12 (21.05)	Ref.	Ref.
Yes	469	33 (7.04)	0.28 [0.14–0.59]	0.31 [0.14–0.68]
Cluster area				
Cluster I	249	22 (8.84)	Ref.	Ref.
Cluster II	277	23 (8.30)	0.93 [0.51–1.72]	0.70 [0.34–1.44]

The main place of malaria blood examination and treatment was malaria post and malaria clinic (88.89%). Over 50% of these individuals with SRM also reported having waited for 2–3 days from onset fever to blood examination and treatment (51.11%) (Table 5). Almost 36% reported waiting > 3 days to seek diagnosis and treatment for malaria.

**Table 5:** Treatment behaviour among individuals with SRM (n = 45)

Treatment behaviours	n	%
Testing and treatment place		
MPs or MCs	40	88.89
Hospital	5	11.11
Duration from onset of fever to treatment		
Within a day (days)	6	13.33
2–3	23	51.11
> 3	16	35.56

## Discussion

The results from this analysis show that HPM among individuals living in malaria hotspots along the Thai–Myanmar border are frequent and also revealed seasonal, socio-economic, and demographic correlates of those movement patterns. This analysis also showed associations between participant characteristics, movement types, and SRM. This information is important for ongoing control and elimination efforts for the overall region.

This analysis showed that trips were more common in the dry season, which is consistent with seasonality and synchronicity of HPM that has previously been reported [22, 25]. Travel during the rainy season is hampered by heavy rains and poor road conditions. Travel among the mostly agricultural populations in this region is also heavily dependent on the agricultural seasons, which are largely driven by the rainy season [22, 26, 27]. There is also a consistent second peak of malaria each year in some parts of the Thai–Myanmar border (evident in Fig. 2), occurring during the dry season (December to January) when travel was more frequent [28]. Infections could be acquired during travel and introduced to the village upon return. Furthermore, parasites from within villages can be exported via travel.

Travel to Myanmar has been considered a risk factor for malaria infection, given that this region in particular has had little health infrastructure (a result of decades of conflict) and therefore higher burdens of malaria [28]. In this analysis, trips to Myanmar were more common among villagers in Cluster I (Tha Song Yang District of Tak Province). The study villages in Cluster I are located immediately adjacent to Myanmar (Fig. 1), and many of the individuals living in this part of Thailand have strong cross-border community and family ties, perhaps especially for those who have relatively few occupational options on the Thai side of the border [14, 29].

Malaria in Southeast Asia (SEA) has also frequently been associated with forests [4, 12, 15], though few studies have shown an empirical relationship. Forest visits among participants in this study were more common among 20 to 39 year olds, males, individuals with low income, low education, and especially among individuals with forest-related occupations. Perhaps especially for individuals with low socio-economic status, forest products offer a means to supplement household food or economic needs [30, 31]. Forest visits may therefore be related to both socio-economic status and to risk of malaria infection in this part of SEA [4, 11, 14].

There were several statistically significant predictors of malaria infection at both the individual and household-level. Individuals who made trips into forested areas during the dry season were more likely to report malaria infections. Individuals who made frequent trips in the dry season in general (i.e. within Thailand or to Myanmar, either overnight or daytrips), were more likely to also report a malaria infection. Individuals who owned and reported using bed nets and those living in elevated houses (Fig. 7) were also less likely to report having a malaria infection. This finding with regard to elevated houses is in line with previous research that suggests mosquito vectors are more common inside ground-level houses than in houses on stilts metre [32–36].

This analysis also suggests that a sizeable proportion (36%) of individuals with malaria infections wait 3 or more days before seeking treatment after the onset of fever. Such delays in treatment can contribute to ongoing transmission, with gametocyte production occurring over the duration of an infection and with increased cumulative exposure to mosquito bites over longer periods of time.

Malaria in the GMS is frequently considered to be more common in adult males. In this analysis there was no significant difference by gender (detailed data by village and gender are provided in Additional file 1). There are several potential reasons for this finding. The infections in this study were self-reported and there were more female participants than males. It is possible that some males who were not present in the villages during the study period would have also reported having malaria infections. The relative number of participants reporting infections was also small (total of 45), and in a larger survey there might have been a detectable difference by gender. It is also possible that the lack of gender difference in SRM is indicative of the real situation in these study villages. When malaria clusters in adult males, it generally indicates exposure to infectious vectors outside of the home village; for example in forests, plantations, agricultural fields, or potentially in other villages that have been visited and act as a reservoir for parasites. Conversely, if a home village is acting as a parasite reservoir, and transmission occurs in village, then infections tend to be more common in both males and females, with demographic (i.e. age and gender) patterns being influenced by village population structure, transmission intensity, and immunity. While most aggregate studies and reports from this region show clustering of malaria in adult males, there are several studies showing little or no difference between males and females [15, 29]. The villages in this study were purposely selected because of their malaria burden and therefore may have different age and gender patterns when compared to areas with less malaria.

There are several limitations to this study. This study attempted to measure the general HPM patterns in malaria hotspots or clusters using community-based surveys and interviews among villagers in these clusters. Therefore, malaria cases used in this analysis were self-reported. It is possible that there is some bias in SRM. However, there are clinics in each of the study villages that regularly diagnose and treat malaria, and a malaria diagnosis is unlikely to have been poorly remembered or forgotten [37]. Other participants may have had asymptomatic infections that were never diagnosed. It is therefore possible that malaria is underestimated in this analysis. Finally, travel was recorded through cross-sectional surveys for a portion of the year (one dry season month and one wet season month). Movement patterns in other months may differ from what was recorded in this study.

## Conclusion

The results from this study suggest that HPM in these malaria hotspots is common, dynamic, and varies by season. Future work should look into the potential importance of the HPM patterns with regard to malaria dynamics in this region. An understanding of HPM (including seasonal patterns in HPM) and concurrent malaria dynamics is important for consideration of targeted public health interventions. Several potential targeted interventions warrant consideration in order to reduce new infections and prevent re-importation of malaria. Vector based approaches, such as residual spraying, using bed nets or insecticide-treated clothing, could help to disrupt transmission in active foci [38, 39]. In addition, a better understanding of mosquito vector behaviour, for example peak biting times for each vector species, could help inform public health approaches. Coverage of diagnosis and treatment centres should be broad, maintained in areas bordering malaria hotspots (even when cases are few), and available to all febrile individuals. In areas with high proportions of asymptomatic infections, other interventions (such as chemoprophylaxis or targeted mass drug administration) may be warranted [40] in order to achieve malaria elimination.

## Additional files

**Additional file 1.** Histogram of number of days spent in trips.

**Additional file 2.** Counts of people with SRM by gender and village.

## Abbreviations

AOR: adjusted odds ratio
CI: confidence interval
GMS: Greater Mekong Sub-region
HPM: human population movement
IRS: indoor residual spraying
OR: odds ratio
SEA: Southeast Asia
SRM: self-reported malaria
THB: Thai Baht
