# Supplementary material for: Human population movement and behavioural patterns in malaria hotspots on the Thai–Myanmar border: implications for malaria elimination
Source: Malar J. 2019 Mar 8;18:64. doi: 10.1186/s12936-019-2704-3 (PMC6408830; doi:10.1186/s12936-019-2704-3)
Supplement: Supplementary file 1 — Additional file 1. Histogram of number of days spent in trips. [file 12936_2019_2704_MOESM1_ESM.pdf]

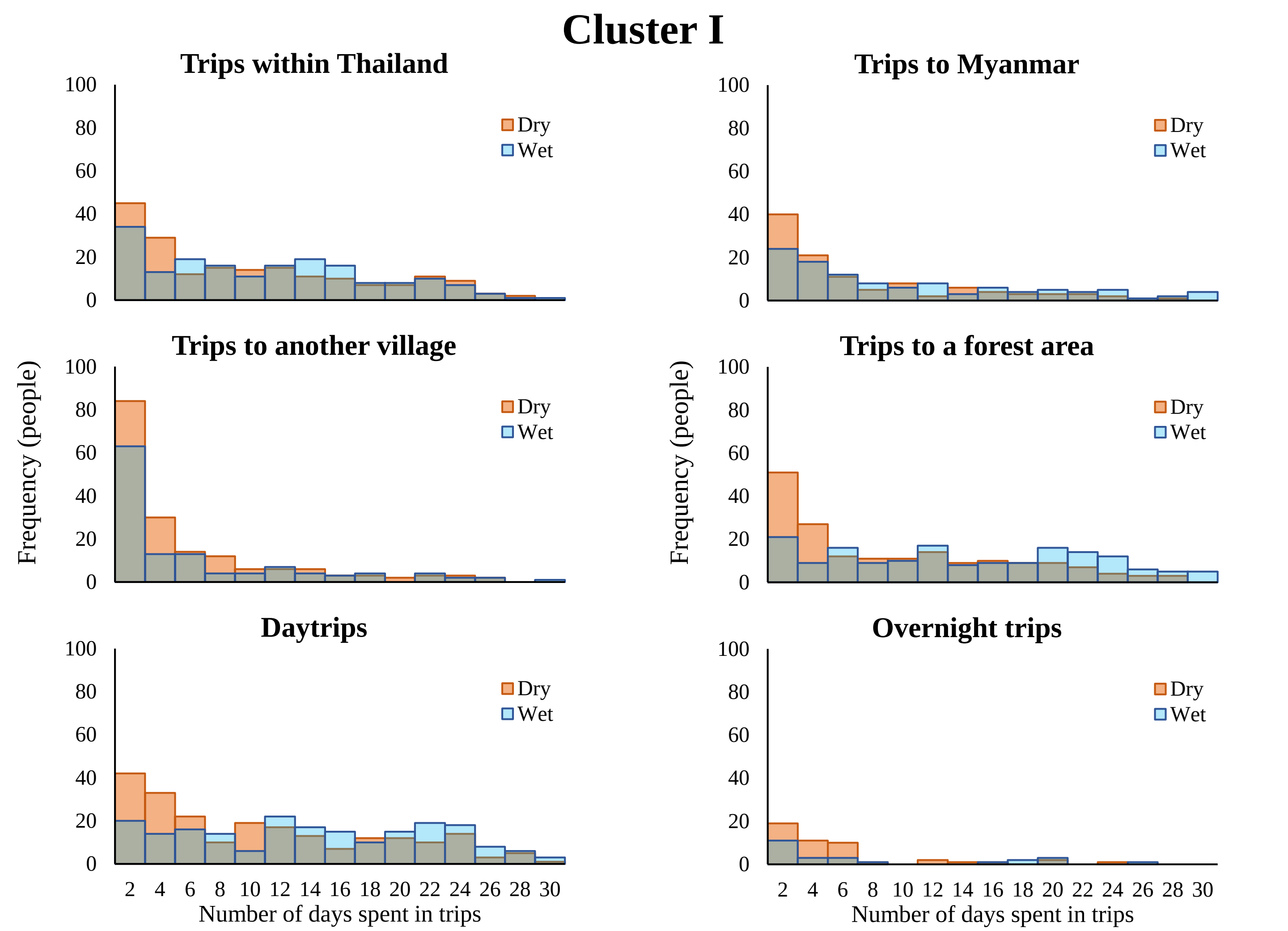
Cluster I
Trips within Thailand
Trips to Myanmar
Trips to another village
Trips to a forest area
Daytrips
Overnight trips
Dry
Wet
Frequency (people)
Number of days spent in trips

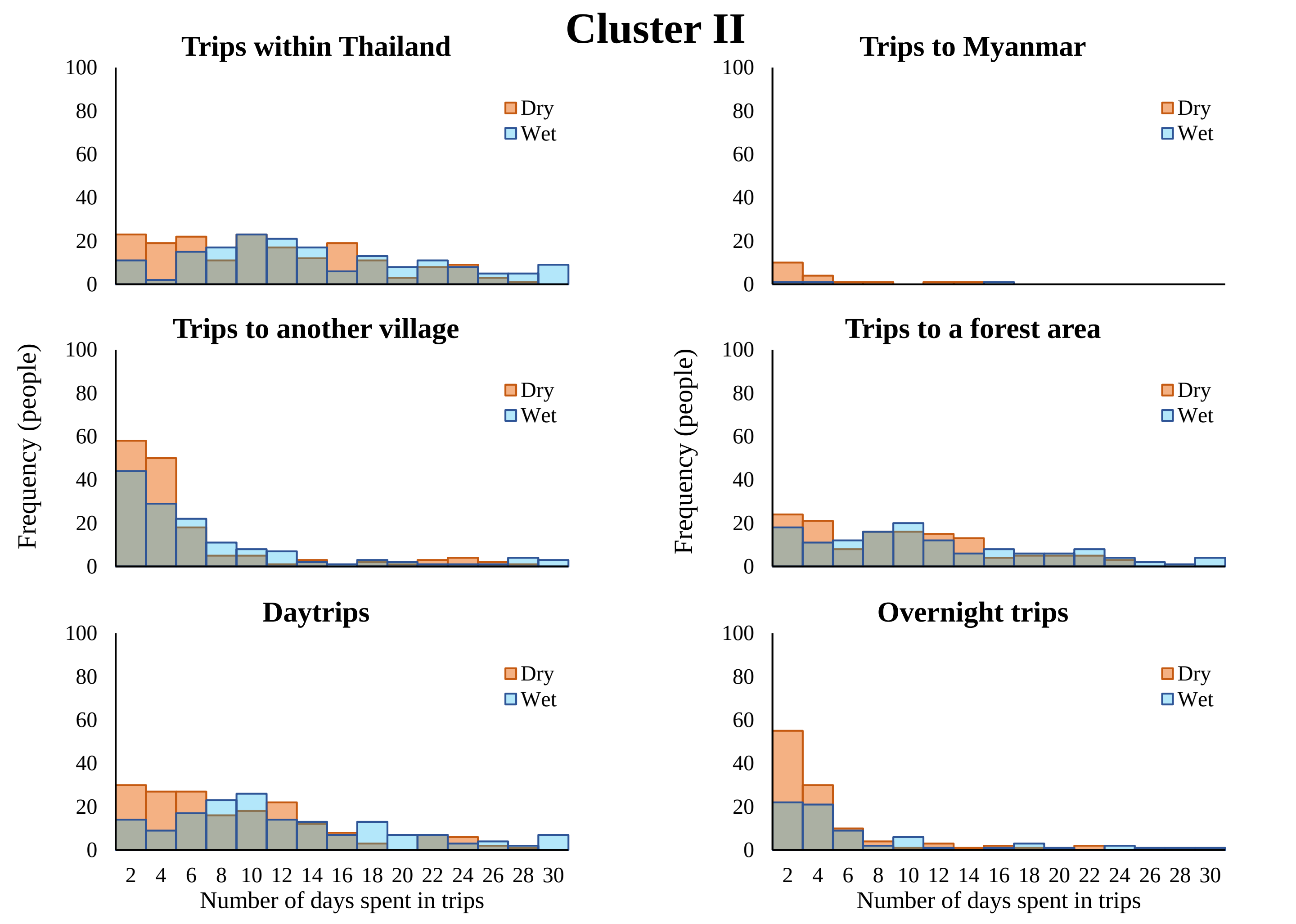
Cluster II
Trips within Thailand
Trips to Myanmar
Trips to another village
Trips to a forest area
Daytrips
Overnight trips
Dry
Wet
Frequency (people)
Number of days spent in trips
