# Supplementary material for: Human population movement and behavioural patterns in malaria hotspots on the Thai–Myanmar border: implications for malaria elimination
Source: Malar J. 2019 Mar 8;18:64. doi: 10.1186/s12936-019-2704-3 (PMC6408830; doi:10.1186/s12936-019-2704-3)
Supplement: Supplementary file 2 — Additional file 2. Counts of people with SRM by gender and village. [file 12936_2019_2704_MOESM2_ESM.docx]

Table S1 Counts of people with SRM by gender and village

| **SRM status** | **n** | **Gender** | |
| --- | --- | --- | --- |
|  |  | **Male (%)** | **Female (%)** |
| **V1: SO** |  |  |  |
| No | 112 | 34 (30.4) | 78 (69.6) |
| Yes | 13 | 7 (53.8) | 6 (46.2) |
| **V2: KMN** |  |  |  |
| No | 115 | 40 (34.8) | 75 (65.2) |
| Yes | 9 | 5 (55.6) | 4 (44.4) |
| **V3: SNP** |  |  |  |
| No | 124 | 58 (46.8) | 66 (53.2) |
| Yes | 15 | 7 (46.7) | 8 (53.3) |
| **V4: WKD** |  |  |  |
| No | 130 | 54 (41.5) | 76 (58.5) |
| Yes | 8 | 3 (37.5) | 5 (62.5) |
